# The effectiveness of sacral lateral branch radiofrequency neurotomy for posterior sacroiliac joint complex pain in patients selected by dual sacral lateral branch blocks; A real-world cohort study

**DOI:** 10.1016/j.inpm.2024.100442

**Published:** 2024-10-30

**Authors:** Austin Boos, Amanda Cooper, Brook Martin, Robert Burnham, Allison Glinka Przybysz, Aaron M. Conger, Zachary L. McCormick, Taylor R. Burnham

**Affiliations:** a Department of Physical Medicine and Rehabilitation, University of Colorado, Aurora, CO, USA; b Department of Physical Medicine and Rehabilitation, University of Utah, Salt Lake City, UT, USA; c Department of Orthopedics, University of Utah Salt Lake City, UT, USA; d Division of Physical Medicine and Rehabilitation, Department of Medicine, University of Alberta, Edmonton, AB, Canada; e Central Alberta Pain and Rehabilitation Institute, Lacombe, AB, Canada; f Vivo Cura Health, Calgary, AB, Canada

**Keywords:** Radiofrequency ablation, Sacroiliac joint pain, Dual lateral branch block

## Abstract

**Background:**

Previous study of spinal neurotomy procedures indicates that stringent block selection improves outcomes. However, this pattern is not established for sacral lateral branch radiofrequency neurotomy (SLBRFN). Few SLBRFN studies have used stringent block selection criteria such as ≥80 % pain reduction following dual sacral lateral branch blocks (SLBB).

**Objective:**

Evaluate the effectiveness of SLBRFN in patients with ≥80 % pain relief following dual SLBBs.

**Methods:**

Retrospective single-arm cohort study of consecutive patients from two Canadian musculoskeletal and pain clinics who underwent first-time SLBRFN after report of ≥80 % pain relief following dual diagnostic SLBBs. Patients were identified by electronic medical record query between 2016 and 2022. The primary outcome was the proportion of individuals with a ≥50 % reduction in the numeric pain rating scale (NPRS) score three months after SLBRFN. Secondary outcomes included the proportion of responders achieving the minimal clinically important difference (MCID) on the pain disability quality-of-life questionnaire (PDQQ), and the duration and mean percentage of pain relief among those with recurrent symptoms after a successful SLBRFN.

**Results:**

Of the 70 participants included, 32 (45.7 %; 95 % CI = 34.6–57.3) reported a ≥50 % reduction in NPRS, and 35 (50.0 %; 95 % CI = 38.6–61.4) achieved the MCID on the PDQQ at 3-months. Among the 17 patients who reported a return of symptoms, the mean duration of relief was 8.0 ± 3.5 months, and the mean percentage of pain relief was 77.9 % ± 16.4 %. Logistic regression models revealed that the use of multi-tined RF probes and lower patient BMI were associated with treatment success.

**Discussion/conclusion:**

SLBRFN reduced pain and disability in approximately 50 % of patients at 3 months when selected using relatively restrictive selection criteria. Treatment success was associated with multi-tined RF probe type and lower patient BMI. Larger prospective studies assessing long-term outcomes are needed to further evaluate the impact of different selection criteria and techniques on SLBRFN effectiveness.

## 1 Introduction

The sacroiliac joint complex (SIJC) consists of the sacroiliac joint (SIJ) and its capsule, along with an extraarticular network of surrounding ligaments or syndesmosis [1]. The intraarticular portion of the SIJC is innervated anteriorly by the rami of the lumbosacral nerve roots. In contrast, the extraarticular portion is innervated posteriorly primarily by the lateral branches of the posterior rami of S1-S3 (the “sacral lateral branches”) with occasional contributions from the L5 dorsal rami and S4 sacral lateral branch [2,3]. The dual innervation of the SIJC makes diagnosis and subsequent treatment of SIJC pain challenging, as pain may arise from the joint capsule, the extraarticular structures, or a combination of the two. Although the intraarticular portion has long been identified as a potential source of posterior pelvic girdle pain, with an estimated incidence of 10–33 % [4], the incidence of SIJC pain arising from extraarticular structures remains unknown.

Traditionally, the primary interventional treatment for SIJC pain has been intraarticular steroid injections into the SIJ. However, radiofrequency neurotomy of the sacral lateral branches (SLB) offers an alternative treatment for SIJC pain with the potential to provide longer-lasting pain relief compared with intraarticular steroid injections [5,6]. Previously published randomized controlled trials indicate that sacral lateral branch radiofrequency neurotomy (SLBRFN) treatment success rates (defined as ≥50 % pain relief) are between 43-64 % and 53–57 % at 3 and 6 months, respectively [7]. This is comparable to the success rates of radiofrequency neurotomy procedures used to treat other axial pain generators, such as the cervical and lumbar zygapophyseal joints [8,9].

Although SLBRFN provides similar treatment outcomes to other RFN modalities, it differs in that there is no established consensus on the optimal block paradigm for selecting patients for this procedure. While the majority of studies to date have used intraarticular sacroiliac joint injections to select patients [7], this selection paradigm lacks concept validity in that SLBRFN exclusively targets extraarticular SLB fibers, with no direct effect on sensory innervation of the anterior portion of the SIJ. In contrast, SLB blocks (SLBBs) possess face and construct validity when selecting patients for SLBRFN. In addition, there remains no consensus regarding the optimal cutoff value of pain relief for SLBBs to be considered successful. More stringent block selection criteria of ≥80 % pain reduction following dual comparative blocks have been explored for RFN of other anatomic structures [10]; however, to our knowledge, no study has directly examined SLBRFN outcomes in patients selected using this prognostic block paradigm. As such, this retrospective cohort study explores the effectiveness of SLBRFN in patients reporting ≥80 % pain relief following dual SLBBs.

## 2 Methods

### 2.1 Data collection

This study was conducted at two Canadian musculoskeletal pain management clinics. The protocol was approved by the Conjoint Health Research Ethics Board at the University of Calgary (Ethics ID#: REB20-0355). The electronic medical records of 572 consecutive patients who underwent SLBRFN between 2016 and 2022 were reviewed. Data extraction was performed by two authors (R.B. and A.A.). Patient inclusion criteria were (a) age 18–80 years (b) with clinical presentation compatible with sacroiliac joint complex pain (pain below L5, positive Fortin finger test, and/or positive SI joint provocative test(s)), refractory to conventional conservative treatments, (c) having undergone two image-guided SLBBs with at least 80 % pain reduction followed by (d) first-time SLBRFN. To meet the block selection criteria, patients needed to report a reduction in pain of 80 % or more on the numeric pain rating scale (NPRS) at any 30-min interval within 6 h following SLBBs, compared to their pre-procedure pain score. Exclusion criteria were (a) undergoing any spine intervention (i.e., epidural or zygapophyseal joint steroid injection) between SLBRFN and follow-up or (b) having the SIJ procedure performed concurrently with any additional lumbar spine interventions. Additional data collected from the electronic medical records included patient age, gender, body mass index (BMI), smoking status, employment status, exercise status, the laterality of procedure, radiofrequency cannula type, duration of index pain, NPRS pain scores before and after SLBB and SLBRFN, and Pain Disability Quality-of-Life Questionnaire- Spine (PDQQ-S) scores before and after SLBRFN.

### 2.2 Sacral lateral branch block technique

All SLBBs were performed using a combination of ultrasound and fluoroscopic guidance previously described by Burnham et al. [11]. Patients were not treated with sedation or anxiolysis during the SLBBs. To begin, a curvilinear or linear ultrasound transducer (dependent on patient body habitus) was placed over the inferior aspect of the sacrum in the transverse plane. After identifying the sacral cornua, the transducer was translated superolateral until the S4 dorsal foramen and S4 transverse sacral tubercle were visualized. The transducer was then translated cephalad until a similar view of the next dorsal foramen and transverse sacral tubercle at S3 was obtained. The skin overlying the S3 transverse sacral tubercle was then marked. Similarly, the S2 and S1 transverse sacral tubercles were identified, and the skin overlying them was marked. A vertical line connecting the three skin markings was created, representing the course of the lateral sacral crest. The vertical line was then marked with intersecting horizontal lines at 1-cm intervals. A 25-gauge needle was inserted at each location where the horizontal lines intersected the vertical line. Each needle was then advanced under fluoroscopic guidance until the tip contacted the sacral periosteum. A total of 0.5 mL of local anesthetic (2 % lidocaine or 0.5 % bupivacaine) was then injected at each site. Patients were then given a pain diary to record an NPRS pain score at 30-min intervals for 6 h following the blocks. If a patient reported ≥80 % pain reduction in the 6 h following blocks, they underwent a second round of SLBBs, performed no sooner than one week from the first series of blocks. If patients reported at least 80 % pain reduction following both sets of SLBBs, they were eligible to proceed to SLBRFN.

If less than 80 % pain relief was achieved within the first 6 h after initial SLB blocks, the L5 dorsal ramus was added to the S1–S3 lateral branch nerves for the second set of SLBBs. The option to add the L5 dorsal ramus in patients with suboptimal response rather than for all patients is based on cadaveric evidence showing the L5 dorsal ramus contributes to SIJ innervation in less than 10 % of cases [2,4,11] All blocks were placed at the periosteal level only rather than multi-depth based on the findings of recent cadaveric dissections confirming that the lateral branch nerves run exclusively along the periosteum [2].

### 2.3 Sacral lateral branch radiofrequency neurotomy techniques

Patients were positioned prone on a standard fluoroscopy table without sedation or anxiolysis. Sacral lateral branch radiofrequency neurotomy (SLBRFN) was performed using one of two techniques. One technique was performed by placing pairs of 18-gauge three-tined 5-mm active tip cannulae (Diros RF Trident, Markham, ON, Canada) in a bipolar configuration under fluoroscopic guidance. The cannulae were placed perpendicular to the sacral periosteum, at locations 1.5 cm apart along the lateral sacral crest between the S1 and S3 sacral tubercle. Three or four conventional bipolar lesions were then created (corresponding to 6–8 total cannula placements) based on the anatomical distance between the S1 and S3 sacral tubercles. The alternative technique included a 16 g/longitudinal ultrasound-assisted procedure that placed a 16 g monopolar cannula with a 2 cm curved active tip at 3 sites along the longitudinal axis of the lateral sacral crest as previously described [11]. Both techniques utilized a 30-s ramp-up period followed by 2 min of traditional thermal lesioning conducted at a temperature of 80 °C.

### 2.4 Data analysis

Data analysis included descriptive statistics and logistic regression analysis, which involved the calculation of odd ratios (ORs) and their corresponding 95 % confidence intervals (CIs). The primary outcome was the proportion of patients who reported at least 50 % pain reduction in NPRS pain score at 3-month follow-up compared with their pre-SLBRFN score. The secondary outcome was the proportion of patients who met the minimal clinically important difference (MCID) on the PDQQ-S questionnaire, a validated six-question patient-reported outcome measure specifically designed for use in the minimally invasive interventional spine care field [12]. The MCID value for this questionnaire has been previously determined to be a 17-point decrease [13]. In patients reporting a return of index pain, retrospective percentage pain relief and duration were also analyzed. Covariates included in the logistic regression models were age, gender, BMI, smoking status, exercise status, employment status, cannula type, and laterality of procedure (unilateral or bilateral). Individuals with missing data for one or more variables in the logistic regression model were automatically excluded from the analysis.

## 3 Results

Of the 572 consecutive patients identified by a database query, 70 met the inclusion criteria. Patient demographic, clinical, and procedure-related variables are described in Table 1. Of the included participants, the mean age was 60.6 ± 13.2 years, and 24.3 % were male. Most patients (82.8 %) underwent SLBRFN using the three-tined cannula. At 3-month follow-up, thirty-two patients (45.7 %; 95 % CI = 34.6–57.3) reported a ≥50 % reduction in NPRS pain score, and 35 patients (50.0 %; 95 % CI = 38.6–61.4) achieved the MCID on the PDQQ-S. Seventeen patients returned for repeat SLBRFN when their pain returned. The mean duration of pain relief was 8.0 ± 3.5 months, and the mean magnitude of pain relief during that period was 77.9 ± 16.4 % (Table 2).

**Table 1:** Patient demographic, clinical, and procedural variables (*N* = 70).

Variable	Frequency (%)
**Gender**	
Male	17 (24.3)
Female	53 (75.7)
**Smoker**	
Yes	8 (15.7)
No	43 (84.3)
*Missing*	*19*
**Exercise**	
Yes	19 (38.8)
No	30 (61.2)
*Missing*	*21*
**Working**	
Yes	18 (39.1)
No	8 (17.4)
Retired	20 (43.5)
*Missing*	*24*
**Workup**	
Internal	50 (71.4)
External	20 (28.6)
**Clinic**	
Practice 1	15 (21.4)
Practice 2	55 (78.6)
**Needle**	
16 g	11 (17.2)
Trident	53 (82.8)
*Missing*	*6*
**Laterality**	
Unilateral	30 (42.9)
Bilateral	40 (57.1)
**Age in years** (*n* = 70); mean (SD)	60.6 (13.2)
**Body mass index in kg/m**^**2**^ (*n* = 48); mean (SD)	30.0 (6.2)
**Pain duration in yr** (*n* = 48); mean (SD)	8.9 (10.1)

**Table 2:** Primary and secondary study outcomes.

Outcome Variable	n (%)	95 % CI
**≥ 50 % NPRS reduction** (*N* = 72)		
Yes	32 (45.7)	34.6, 57.3
No	38 (54.3)	42.7, 65.4
**≥ 17-point PDQQ reduction** (*N* = 72)		
Yes	35 (50.0)	38.6, 61.4
No	35 (50.0)	38.6, 61.4
**Retrospective Percentage Pain Relief** (*N* = 17; mean [SD])	77.9 (16.4)	
**Retrospective Duration of Improvement** in months (*N* = 17; mean [SD])	8.0 (3.5)	

Abbreviations: CI = confidence interval; NPRS = numeric rating scale; PDQQ = Pain Disability Quality-of-Life Questionnaire; SD = standard deviation.

Logistic regression models for ≥50 % NPRS reduction and MCID on the PDQQ-S are summarized in Table 3. None of the selected variables were significantly associated with ≥50 % NPRS reduction. Using a perpendicularly aligned three-tined cannula (versus a longitudinally aligned conventional 16-gauge cannula) during SLBRFN was associated with significantly higher odds of meeting the MCID on the PDQQ-S (OR = 12.91; 95 % CI = 1.18–141.35). Conversely, patients with a greater BMI were less likely to meet the MCID on the PDQQ-S by 15 % (OR = 0.85; 95 % CI = 0.73–0.98).

**Table 3:**
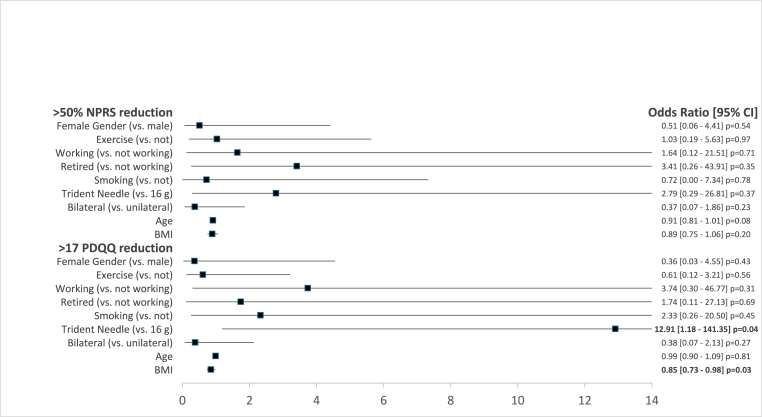
Forest plot of logistic regression models on ≥50 % NPRS reduction and ≥17-point PDQQ reduction.

^a^*N* = 38; χ^2^ (9) = 11.74; *p* = 0.23; Pseudo *R*^2^ = 0.29.

^b^*N* = 38; χ^2^ (9) = 9.18; *p* = 0.42; Pseudo *R*^2^ = 0.21.

Abbreviations: CI = 95 % confidence interval; NPRS = numeric rating scale; PDQQ = Pain Disability Quality-of-Life Questionnaire.

## 4 Discussion

Sacral lateral branch radiofrequency neurotomy (SLBRFN) is an effective intervention for alleviating pain and improving function in patients with SIJC pain. However, a clear consensus regarding the optimal patient selection paradigm has not been established**.** Our study examined the success rate of SLBRFN in patients selected based on a more stringent block selection paradigm consisting of two sets of single-depth SLBBs with a cutoff of ≥80 % pain reduction. We present the largest retrospective cohort published to date exploring this specific block paradigm in patients before SLBRFN. Our findings revealed that approximately half of patients treated with SLBRFN reported clinically significant improvements in pain and function at three-month follow-up. Additionally, we identified the utilization of a bipolar configuration of perpendicularly aligned three-tined radiofrequency (RF) cannula type and lower patient BMI as predictors of treatment success.

Several recent systematic reviews have described the effectiveness of SLBRFN for the treatment of SIJC pain. In 2015 King et al. reported a pooled responder rate, defined by ≥ 50 % pain reduction, of approximately 50 % at three months post-procedure [14]. A more recent systematic review by Lee et al. in 2023 reported similar success rates ranging from 43 % to 64 % based on the randomized controlled studies included in the review. In addition to affirming the efficacy of SLBRFN, the reviews by Lee et al. and King et al. underscore the significant heterogeneity that exists in the literature with respect to patient selection methods and SLBRFN technique. For instance, most studies to date have relied on intraarticular SIJ blocks for patient selection. However, this practice lacks concept validity because the intraarticular and extraarticular portions of the SIJC have separate innervation pathways. SLBRFN does not directly affect nociception from the anterior portion of the SIJC.

Among the studies that utilized SLBBs for patient selection, there is significant heterogeneity in regard to the optimal number (1 *versus* 2), location (single site *versus* multisite), depth (single depth *versus* multi-depth), and pain relief cutoff value. Drawing from available lumbar spine data, two sets or “dual comparative” blocks are superior to single blocks due to their lower rate of false positives [15]. Due to variability in the anatomic location of the S1-S3 SLBs as they exit the corresponding dorsal foramen, multisite and multi-depth blocks are often considered superior to single-site, single-depth blocks as they possess a higher probability of completely anesthetizing the SLBs. While we agree that multisite blocks are theoretically preferable over single-site blocks, both clinical and cadaveric literature reports have shown that single-depth blocks are equivalent to multi-depth blocks [2,16]. Finally, the optimal pain relief cutoff threshold for a positive SLBB response remains debated. While some advocate for more stringent cutoffs (≥80 %) to reduce the rate of false positives, others have argued that stringent cutoffs limit patient access to potentially beneficial treatment and, as such, advocate for more modest thresholds of ≥50 % pain reduction [10,17]. Although different block selection cutoffs have not been directly compared for SLBRFN, they have been explored in medial branch RFN procedures in the cervical and lumbar spine [8,9]. These investigations report no significant difference between more stringent criteria (>80 % pain reduction) and less stringent criteria (>50 % pain reduction) when the outcome of interest is the proportion of patients with >50 % pain reduction following RFN. However, when the outcome is the proportion of patients with >80 % or 100 % pain relief, the more stringent criteria are superior to less stringent criteria, which is important to contextualize with patients finding 30 % or greater pain relief as meaningful [18]. While block selection criteria before SLBRFN have been indirectly assessed most recently in the systematic review conducted by Lee et al., to date no studies have specifically investigated patients selected using dual comparative SLBBs with an ≥80 % pain reduction cutoff, hence the need for our study.

While no studies have used this criterion, two studies, Loh et al. and Patel et al. offer the most analogous comparisons to our study albeit both are limited by differences in study design [19,20]. Loh et al. conducted a prospective, single-arm cohort study that examined ultrasound-guided bipolar RFN of the S1-S3 SLBs using a multi-tined cannula in patients selected via dual SLBBs with a cutoff of ≥50 % pain reduction. Extrapolation from their data suggests that the 3-month success rate would likely fall between 61 % and 41 %, which were the rates reported at 2- and 6-month follow-up, respectively. Patel et al. conducted a sham-controlled RCT examining cooled RFN using a monopolar cannula of the L5 dorsal rami and S1-S3 SLBs in patients selected with dual SLBBs using a ≥75 % pain reduction cutoff. The authors reported a 3-month success rate of 53 % (95 % CI = 36–70 %). While we hypothesized that the use of more stringent block selection criteria would improve SLBRFN outcomes via a reduction in false positives, we observed a success rate of approximately 50 % in the present study, which is comparable to those reported by both Loh et al. and Patel et al., as well as other randomized controlled trials of SLBRFN [7]. As such, it remains unclear whether more stringent selection criteria improve patient success rates.

One additional notable finding from our study is the association between certain procedural and patient factors and treatment outcomes. We found that the use of the three-tined RF cannula was predictive of successful outcomes for function (defined here as meeting the MCID on PDQQ-S) but not for pain relief (≥50 % pain reduction). This may represent an increased ability to capture the SLB nerves, as both the three-tined cannula lesion and perpendicular bipolar lesioning technique produce large lesion sizes and can probably better accommodate undulations of the dorsal sacrum in comparison to the longitudinally aligned 16-gauge monopolar cannula. While several cannula types and lesioning techniques have been described in the literature and are available commercially, only cooled monopolar and bipolar strip lesioning techniques have been anatomically validated [2,21]. In addition to cannula type, patient BMI was also inversely associated with treatment success measured by PDQQ. It is unclear why this relationship between low BMI and treatment success was seen. While body habitus can theoretically make the procedure more technically challenging and has been shown to increase procedural fluoroscopy time [22,23], a clear relationship between BMI and pain procedure outcomes has not been definitively established. In fact, prior investigations examining predictors of RFN in other body regions have been mixed in terms of the association between BMI and outcomes [[24], [25], [26]]. As such this finding may be unique to our patient population, and caution should be used when extrapolating to other patient populations or procedures.

While our study contributes valuable insights into SLBRFN effectiveness and helps address the paucity of evidence regarding patient selection paradigms, it bears several limitations. One limitation is the retrospective nature of the study and the relatively small sample size. An additional potential limitation is the amount of missing demographic and treatment variable data. Finally, the lack of long-term follow-up is another limitation of the study. Among the patients who returned for repeat SLBFRN the retrospective mean duration of relief was reported as 8 months during which the mean percentage of pain relief was approximately 80 %. While this indicates that SLBRFN provides durable pain relief, it was only based on 17 patients, and as such firm conclusions about the durability of improvements following SLBRFN cannot be made based on our study.

Future prospective studies with larger sample sizes and longer follow-ups are needed to continue to build the evidence base for the effectiveness of SLBRFN. Ideally, these studies should only use both validated block techniques, multi-site dual comparative SLBBs, and anatomically validated RFN techniques. More crucially, additional prospective or larger-scale retrospective studies are needed to firmly establish optimal block selection criteria and specifically, whether there are any additional benefits in outcomes with using more stringent patient selection criteria of ≥80 % pain reduction.

## 5 Conclusion

SLBRFN demonstrates effective pain and disability reduction at three months in approximately 50 % of patients selected using stringent selection criteria. The use of a three-tined RF cannula type and lower patient BMI predicted treatment success. Larger prospective studies of long-term outcomes are needed to evaluate the impact of different selection criteria and techniques on SLBRFN effectiveness.

## Conflict of interest statement

Zachary L. McCormick, MD serves on the Board of Directors of the International Pain and Spine Intervention Society (IPSIS), has research grants from Avanos Medical, Boston Scientific, Relievant Medsystems, Saol Therapeutics, Spine Biopharma, SPR Therapeutics, Stratus Medical (paid directly to the University of Utah), and also consultancies with Avanos Medical, Saol Therapeutics, Stryker, and OrthoSon. Taylor Burnham, DO received research grant funding from DIROS technology (paid directly to the University of Utah) and does consulting work for Avanos Medical. Aaron Conger, DO received research grant funding from Stratus LLC (paid directly to the University of Utah). There are no other potential conflicts of interest to disclose on the part of any of the other authors.

## Funding

None.

## Declaration of competing interest

The authors declare the following financial interests/personal relationships which may be considered as potential competing interests:

Zachary McCormick, MD reports a relationship with Polytechnic Institute of Santarém that includes: board membership. Zachary McCormick, MD reports a relationship with Avanos Medical Inc that includes: consulting or advisory and funding grants. Zachary McCormick, MD reports a relationship with Boston Scientific Corporation that includes: funding grants. Zachary McCormick, MD reports a relationship with Relievant Medsystems Inc that includes: funding grants. Zachary McCormick, MD reports a relationship with Saol International Ltd that includes: consulting or advisory and funding grants. Zachary McCormick, MD reports a relationship with spine biopharma that includes: funding grants. Zachary McCormick, MD reports a relationship with SPR Therapeutics Inc that includes: funding grants. Zachary McCormick, MD reports a relationship with Stratus Medical that includes: funding grants. Zachary McCormick, MD reports a relationship with Stryker Spine that includes: consulting or advisory. Zachary McCormick, MD reports a relationship with OrthoSon that includes: consulting or advisory. Taylor Burnham, DO reports a relationship with Diros Technology Inc that includes: funding grants. Taylor Burnham, DO reports a relationship with Avanos Medical Inc that includes: consulting or advisory. Aaron Conger, DO reports a relationship with Stratus LLC that includes: funding grants. If there are other authors, they declare that they have no known competing financial interests or personal relationships that could have appeared to influence the work reported in this paper.
